# Ring vaccination and vector control as control strategies for potential yellow fever outbreak in an Asian city

**DOI:** 10.1016/j.idm.2025.07.008

**Published:** 2025-07-20

**Authors:** Guo Jing Yang, Haolong Song, Jue Tao Lim, A. Janhavi, Gregory Gan, Guan Tong, Pei Ma, Nigel Lim Wei Han, Muhammad Hafiz Bin Mohd Aziz, Borame L. Dickens

**Affiliations:** a Saw Swee Hock School of Public Health, National University of Singapore, Singapore; b Lee Kong Chian School of Medicine, Nanyang Technological University, Singapore

## Abstract

**Background:**

Yellow Fever (YF) importation remains an active risk to Southeast Asia. This study aims to determine the effectiveness of vector control and ring vaccination as containment strategies.

**Methods:**

We modelled a YF outbreak in Singapore over 1 year using a metapopulation vector-host spatial model to explore the impact of a potential epidemic and intervention effectiveness. 30 different scenarios were examined by varying the vector to human ratio *m* ([1, 3, 6]), vaccination coverage ([10 %, 50 %, 90 %]) and delay in vaccine rollout ([7, 14, 30 days]), including three non-vaccination scenarios with the vector-to-human ratio m ([1, 3, 6]).

**Results:**

Vector control has a significant protective effect with an 89 % reduction in the cumulative number of exposed cases at Day 365 when lowering *m* from 6 to 1 in the baseline scenario without ring vaccination. Vaccination coverage levels of 90 %, 50 %, and 10 % reduce the cumulative number of exposed cases by 88 %, 56 %, and 12 %, respectively, compared to baseline, when fixing m = 3 and a 7-day rollout delay. A greater number of severe infections and deaths can be mitigated by decreasing the ratio m compared to ring vaccination strategies. The marginal gains in averting the number of infections and deaths are most significant when m is decreased, followed by increased vaccination coverage and reduced intervention delay as R_0_ is proportional to $\sqrt{m}$. This highlights the central role of vector control. Our findings suggested that ring vaccination is effective under lower mosquito-to-human ratios up to 1-week post-detection, with vaccination coverage of at least 50 %. Under these settings, vaccine doses equal to 25 % of the total population are needed to contain the initial outbreak, allowing time to monitor its progress and restock the supply. After that, further interventions where YF has not yet been declared endemic.

**Conclusion:**

Our findings suggested that ring vaccination is effective under lower mosquito-to-human ratios up to 1-week post-detection, with vaccination coverage of at least 50 %. After that, further interventions are required to bring the effective reproduction number *R*_*eff*_ under 1, highlighting the need for rapid response and containment, preparation in the stockpiling of vaccines, and continual suppression of mosquito vector populations when faced with the risk of YF importation and outbreak.

## 1 Introduction

Yellow fever (YF) is a disease endemic to tropical regions of Africa and South America, transmitted by female *Aedes aegypti* mosquitoes (Chen & Wilson, 2020). Symptoms include fever, jaundice and fatigue, although an estimated 55 % of YF cases are asymptomatic, 33 % exhibit mild symptoms and 12 % experience severe disease (Johansson et al., 2014). In severe illness, the disease progresses to a toxic phase, characterized by further fever, nausea, vomiting, epigastric pain and renal insufficiency (Kleinert et al., 2019; Monath, 2001). Mortality is relatively high for those experiencing severe disease at an estimated 47 % due to complications such as internal bleeding and multi-system organ failure (Johansson et al., 2014; Kleinert et al., 2019; Monath, 2005).

An estimated 109,000 severe YF infections and 51,000 YF deaths have occurred in Africa and South America (Gaythorpe et al., 2021). Major outbreaks have been recorded during periods of increased vector breeding. The primary vector is *Aedes aegypti*, which is also a dengue vector (Barnett, 2007). Although dengue cases continue to be recorded across Asian and Pacific countries, with 2 billion people residing in areas occupied by this mosquito vector, YF is largely absent in this region (Lataillade et al., 2020; Ooi et al., 2006). The risk of importation is not, however, null. In 2016, documented YF cases were recorded in China having been imported by returning travellers from Angola (Cui et al., 2017). A total of 11 returning workers were diagnosed (Wilder-Smith & Leong, 2017). Among these, 6 were fatalities, highlighting the severity of the disease and potential challenges for healthcare systems in Asia (Wilder-Smith & Leong, 2017). Singapore, an equatorial city-state where both dengue and *Ae. aegypti* localized breeding are highly endemic, has yet to experience a YF outbreak, which can require more extensive and long-term patient care compared to dengue (Kleinert et al., 2019; Rogers et al., 2006). Such an outbreak is expected to incur hospital capacity strain and substantial fatalities, necessitating the planning of rapidly implementable public health interventions.

Two main control measures can be implemented in Singapore: vaccination and vector control. For vector control, Singapore has a world-leading programme incorporating vector and virological surveillance, management of vector-breeding sites as well as novel strategies such as Wolbachia (Liew et al., 2021). This has led to a substantial reduction in the vector-breeding index, its success evident in the sizeable reduction in the seroprevalence of dengue (Hoffmann et al., 2011; Tan et al., 2019). Specifically for YF, vaccination strategies, however, remain the primary form of intervention for at-risk populations in Africa and South America, as the vaccine provides long-term immunity and is highly efficacious (Shearer et al., 2017). While the YF vaccine is generally safe, there is a risk of adverse events following immunization (AEFI) wherein the vaccine may cause damage to the liver, kidneys or nervous systems at a risk of 0–0.21 in endemic areas and 0.09–0.4 in non-endemic areas (CDC, 2024). Older individuals are at higher risk, meriting careful risk-benefit assessment. This precludes its administration at the population level in Singapore, where YF is not yet in active circulation and about 17 % of the population is over 65.

If travellers from other epidemic sites enter Singapore, ring vaccination strategies may instead be deployed, entailing vaccination of close contacts and those in the proximity of confirmed cases in efforts to prevent ongoing transmission. These have been applied successfully against smallpox and the Ebola virus (Kucharski et al., 2016; Wells et al., 2019). Such a strategy could be advantageous for YF, as it can be rapidly deployed upon detection to control a localized outbreak, negating the need for a population-level vaccination response. To efficiently deploy ring vaccination, vaccine stockpiling is required; however, estimations of stock size are required in advance due to manufacturing lags and potential demand from ongoing YF epidemics. We thus model different ring vaccination coverage levels post-case importation and identification of a case and consider the time required for coverage to be achieved. Examining the impacts of different ring vaccination schemes upon the arrival of YF in Singapore can also assist in tailoring response protocols within policy planning for new or larger epidemics elsewhere.

Thus, this study first estimates the local spread upon case importation within a fine-grained city-level population model previously used for other infectious diseases. Then, it examines the efficacy of ring vaccination and vector control as intervention strategies for curbing transmission in Singapore.

## 2 Methods

### 2.1 Models

We apply a geospatial vector-host transmission model to simulate a YF outbreak, consisting of a vector-host model coupled with a spatial interaction model. In the spatial interaction model, we separate the population into 8619 equally sized hexagons. People travel back and forth between hexagons based on their residential and work/school addresses.

#### 2.1.1 Yellow fever model

**Fig. 1:**
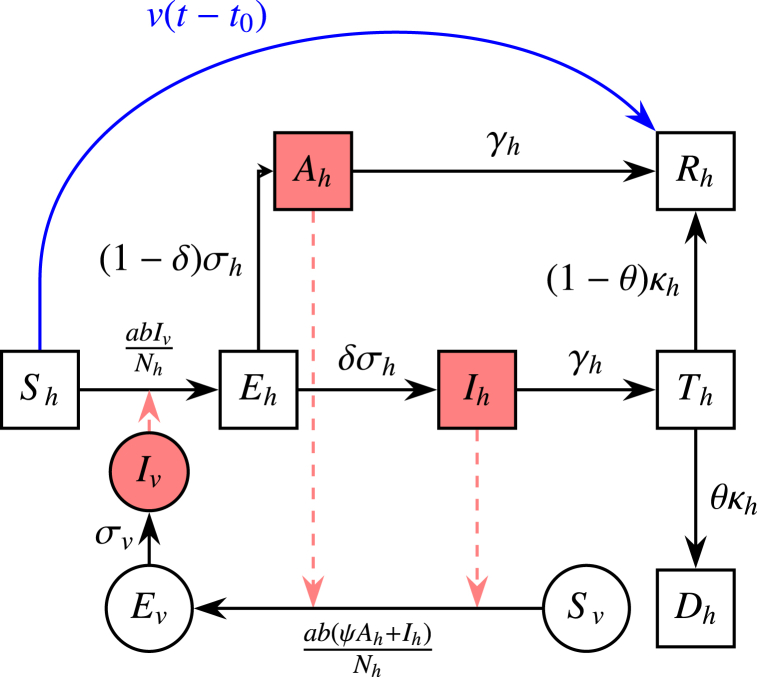
Schematic of vector-host model. Black arrows represent the transition paths of the host/vector's stage, red dashed arrows represent transmission paths, and blue arrows represent the vaccination pathway. Square compartments represent host classes and circle compartments represent vector classes.

The vector-host model is shown in Fig. 1. With this model, we investigate how different intervention strategies affect the severity and size of the outbreak.

In Fig. 1, notations with subscript *h* represent the host classes, and subscript *v* represent the vector classes. *S*_*h*_ represents the number of susceptible individuals, *E*_*h*_ the individuals exposed to YF but not yet infectious, *A*_*h*_ the asymptomatic individuals (i.e., with clinically inapparent symptoms), *I*_*h*_ the severely infected individuals, and *T*_*h*_ the individuals in the toxic stage. Finally, *R*_*h*_ individuals have either recovered from the disease and/or been immunised by vaccination. For vectors (mosquitoes), *S*_*v*_ represents the number of susceptible mosquitoes, *E*_*v*_ the mosquitoes exposed to YF but aren't infectious, and *I*_*v*_ the infected mosquitoes which can transmit the virus to hosts.

There are two main transmission pathways for the host population. The first is

(1) $$S_{h}\to E_{h}\to I_{h}\to T_{h}\to R_{h}\text{.}$$

In this pathway, susceptible hosts are exposed to YF via the bites of infectious mosquitoes (*I*_*v*_), harbour the virus, and move to the exposed stage. They transit to the infected stage and then to the toxic stage. At this stage, the hosts will either die with a probability of case-fatality-ratio (CFR) *θ* or gain lifelong immunity (recovered stage) with probability 1 - *θ*.

The second pathway is

(2) $$S_{h}\to E_{h}\to A_{h}\to R_{h}\text{.}$$

This is the most common transition pathway. Susceptible hosts become exposed to YF by the bites of infectious mosquitoes, harbour the virus and move to the exposed stage. They soon become mildly infected, either exhibiting mild symptoms or being asymptomatic, eventually recovering and obtaining lifelong immunity.

In this model, the asymptomatic (*A*_*h*_) and infected, (*I*_*h*_) hosts can infect mosquitoes if they are bitten. However, the asymptomatic cases have reduced transmissibility ψ compared to the infected cases. Individuals in the toxic stage aren't infectious as they no longer have viremia (Singapore Department of Statistics, 2021) and would be hospitalised.

The system of ordinary differential equations (ODEs) for the vector-host model illustrated in Fig. 1 is as follows:

(3) $$S_{h}^{\prime }=-ab\frac{I_{v}}{N_{h}}S_{h}-\nu \left(t-t_{0}\right)$$

(4) $$E_{h}^{\prime }=ab\frac{I_{v}}{N_{h}}S_{h}-\sigma_{h}E_{h}$$

(5) $$A_{h}^{\prime }=\left(1-\delta \right)\sigma_{h}E_{h}-\gamma_{h}A_{h}$$

(6) $$I_{h}^{\prime }=\delta \sigma_{h}E_{h}-\gamma_{h}I_{h}$$

(7) $$T_{h}^{\prime }=\gamma_{h}I_{h}-\kappa_{h}T_{h}$$

(8) $$R_{h}^{\prime }=\nu \left(t-t_{0}\right)+\gamma_{h}A_{h}+\left(1-\theta \right)\kappa_{h}T_{h}$$

(9) $$D_{h}^{\prime }=\theta_{\kappa_{h}}T_{h}$$

(10) $$S_{v}^{\prime }=B_{v}\left(t\right)-ac\frac{\psi A_{h}+I_{h}}{N_{h}}S_{v}-\mu_{v}S_{v}$$

(11) $$E_{v}^{\prime }=ac\frac{\psi A_{h}+I_{h}}{N_{h}}S_{v}-\sigma_{v}E_{v}-\mu_{v}E_{v}$$

(12) $$I_{v}^{\prime }=\sigma_{v}E_{v}-\mu_{v}I_{v}$$

where *v*(*t*) represents the vaccination rate at time *t*, *t*_0_ is the mean period from receiving vaccination to attaining full immunity and *B*_*v*_(*t*) is the mosquito birth rate. The representation of the remaining parameters and the range of values taken can be found in Table 1.

**Table 1:** Parameter values and units.

Parameter	Notation	Value	Unit/Notes
Mosquito biting rate	*a*	0.3–1.0	Per vector-day
Transmission probability from vector to host	*b*	0.10–0.75	Per bite
Transmission probability from host to vector	*c*	0.30–0.75	Per bite
Host latent period	*σ*_*h*_^−1^	3–6	Days
Vector latent period	*σ*_*v*_^−1^	8–12	Days
Non-severe case relative infectivity	*ψ*	0.1–0.5	–
Host infectious period	*γ*_*h*_^−1^	3–4	Days
Severe case proportion	*δ*	15 %	–
Severe case CFR	*θ*	0 %–50 %	–
Toxic phase duration	*κ*_*h*_^−1^	7–10	Days
Vector lifespan	*μ*_*v*_^−1^	4–35	Days
Mean period from vaccination to full immunity	*t*_0_	21	Days

Our model assumes that the total number of host individuals, *N*_*h*_, is constant:

(13) $$N_{h}=S_{h}+E_{h}+A_{h}+I_{h}+T_{h}+R_{h}+D_{h}=constant\text{.}$$

The total number of female mosquitoes (males do not bite), *N*_*v*_, is related to *N*_*h*_ by the ratio of vector-to-human populations, *m* (Gao et al., 2016; He et al., 2017):

(14) $$N_{v}=m\cdot N_{h}\text{.}$$

For simplicity, we assume *m* is constant and, therefore, *N*_*v*_ is constant. Consequently, the mosquitoes' birth and death rates are equal:

(15) $$N_{v}=S_{v}+E_{v}+I_{v}=constant$$

(16) $$B_{v}\left(t\right)=\mu_{v}\left(S_{v}+E_{v}+I_{v}\right)\text{.}$$

The numeric values for all parameters used in the YF model in Equations (3), (4), (5), (6), (7), (8), (9), (10), (11), (12)) are shown in Table 1. A uniform distribution is assumed in generating values for parameters that have a range. These parameter values and ranges were obtained from previous studies of YF and have been adopted widely in the literature (Andraud et al., 2012; CDC, 2024; Yellow fever; Chikaki & Ishikawa, 2009; Monath, 2008; Zhao et al., 2018).

For parameters related to the host population, *σ*_*h*_^−1^ and *γ*_*h*_^−1^ represent the latent and infectious periods respectively; both are approximately four days. The toxic phase duration, *κ*_*h*_^−1^, is approximately 8 days.

The parameters for vectors were taken from the dengue literature (Andraud et al., 2012; Chikaki & Ishikawa, 2009; Monath, 2008) as it is known that dengue and YF viruses belong to the same family of viruses (i.e., *Flaviviridae*). In urban settings, both viruses are carried by the same vector (i.e., female *Aedes aegypti*). Hence, we follow the literature in assuming they have similar parameter values. The mosquito biting rate (*a*), the transmission probabilities (*b*, *c*) and the vector lifespan *μ*_*v*_^−1^ were taken from two studies of dengue transmission (Andraud et al., 2012; Chikaki & Ishikawa, 2009). The vector latent period *σ*_*v*_^−1^ is approximately 10 days per a 2008 study (Monath, 2008).

#### 2.1.2 Spatial interaction model

**Fig. A.1:**
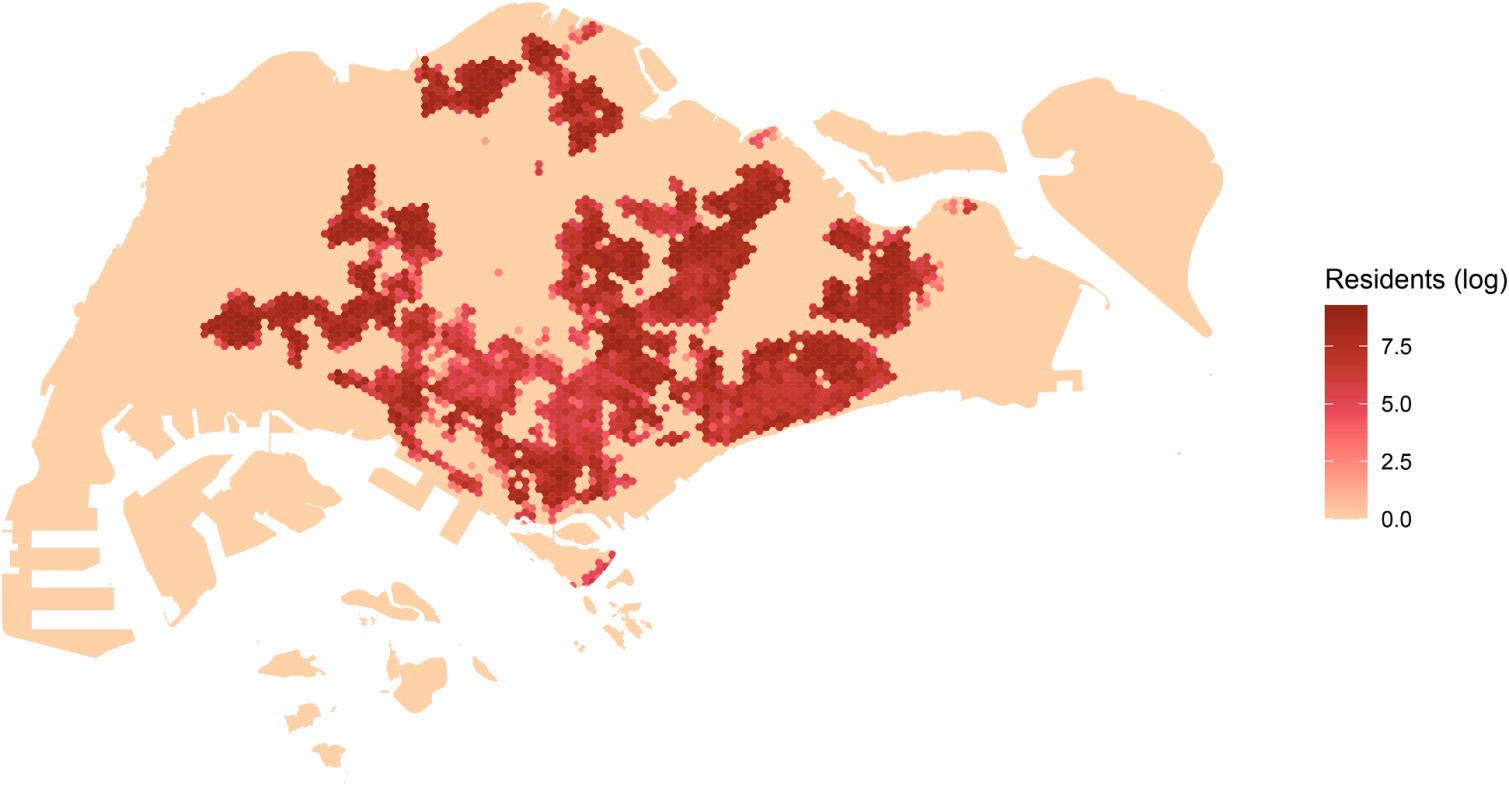
Residents' population density in Singapore in 2023, with log(1+*x*) scale. Notable concentrated areas are Jurong West to the west, Woodlands to the north, and Sengkang and Bedok to the east.

We coupled the vector-host model with a spatial interaction model to gain better insights into the spread of YF and the effects of intervention strategies in Singapore. We divided Singapore into 8,619 equally sized hexagons. Each person was assigned a home hexagon and a work/school hexagon based on their home and work/school postcode. Figures A.1 and A.2 show the geospatial distribution of Singapore's residential and work/school locations, respectively.

### 2.2 Intervention variables

We vary the vaccination coverage level *vc*, delay *d* from a detected infection to the start of vaccination, and ratio of vector (female mosquito) to host populations, *m*. We investigate vaccination coverage levels *vc* of 10 %, 50 % and 90 %, delays *d* of 7, 14 and 30 days, and ratios *m* of 1, 3 and 6. The total number of configurations is 30 (including 3 no-vaccination scenarios). For vaccination strategies, when an infected person is detected in a hexagon, we initiate the ring vaccination (after the set delay), wherein we vaccinate all susceptible host populations in the infected hexagon. The ring vaccination takes three days to complete, and the daily vaccination rate is constant (i.e. for higher vaccination coverage levels, the daily vaccination rate is proportionally higher).

### 2.3 Modelling

More details about modelling initialization, configurations and algorithms can be found in Appendix A.

### 2.4 Reproduction number, R_0_

A full description of the method used to compute *R*_0_ is provided in Appendix B**.**

## 3 Results

### 3.1 Cumulative cases

**Fig. 2:**
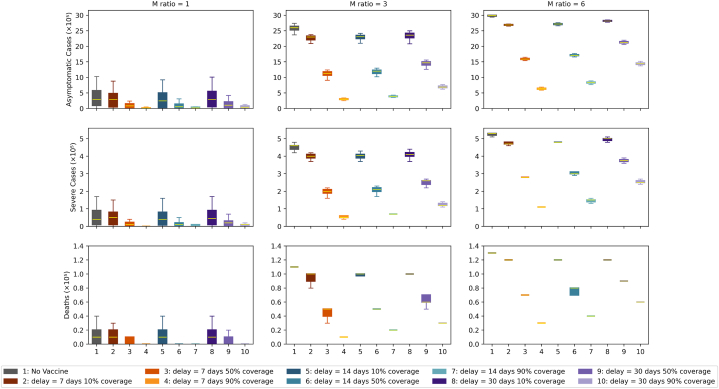
Box plots of the cumulative daily case counts at Day 365 since the outbreak. Outcomes of vaccination strategies under varying mosquito-to-human (M) ratios and vaccination delays or coverage. The columns represent increasing M ratios from left to right: 1, 3, and 6. Rows correspond to different outcome measures: asymptomatic cases (top), severe cases (middle), and deaths (bottom). Orange denotes categories with a 7-day delay, teal denotes categories with a 14-day delay and purple denotes categories with a 30-day delay. Each boxplot within a panel shows the distribution of outcomes under one of ten scenarios, as indicated by the legend, ranging from no vaccination (1) to vaccination with varying delays (7, 14, 30 days) and coverage levels (10 %, 50 %, 90 %).

The box plots of the cumulative numbers of daily cases for each scenario after day 365 are shown in Fig. 2.

#### 3.1.1 Asymptomatic infections

For asymptotic infections, when m = 1 and no intervention is made, the median cumulative case count after day 365 is 285,372 (95 % CI: 4–987,085). This corresponds to 8 % of the total population. When 10 % of the affected population is vaccinated, case counts are similar within 0.1 % across the 7-day, 14-day and 30-day delay scenarios, suggesting no significant effects for 10 % vaccination coverage. The fluctuation in numbers can be explained by stochastic variation. We note that under 10 % vaccination coverage and m = 1, 5 % of simulations resulted in fewer than 30 cumulative asymptomatic cases in the no-vaccination scenario, 2 % under a 7-day delay, 3 % under a 14-day delay, and 3 % under a 30-day delay. When ordered by the upper limit, the cumulative case counts in increasing order are for 7-day, 14-day, 30-day delays, and no intervention. Increasing the vaccination coverage to a higher percentage (50 % and 90 %) decreases the cumulative number of asymptomatic infections. With *m* = 1 and a 7-day delay, increasing the vaccine coverage by 50 % and 90 % decreases the median cumulative numbers of asymptomatic infections by 71 % and 97 % respectively.

**Fig. 3:**
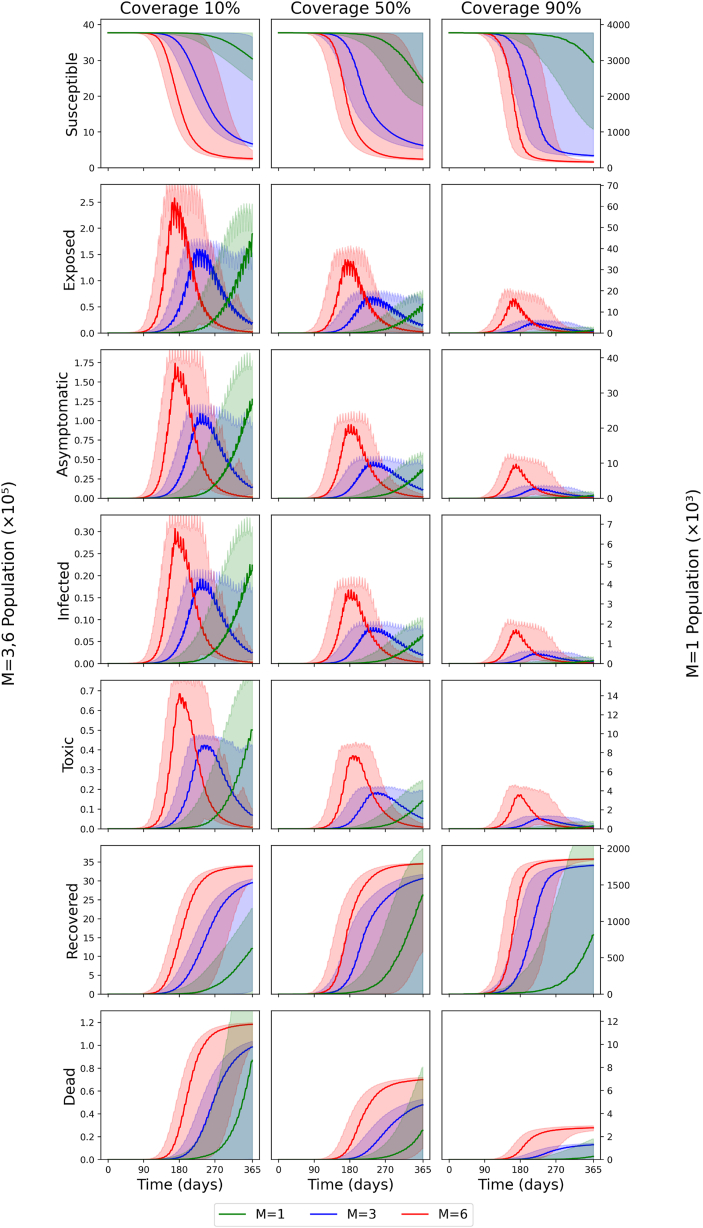
Number of daily active cases for all combinations of vaccination coverage levels and ratios *m* with a fixed vaccine delay (delay = 7 days). Ratios m of 1, 3, and 6 are denoted by green, blue and red, respectively. Darker colours denote the median, and lighter colours denote the 95 % confidence interval. Vaccination coverage increases from left (10 %) to middle (50 %) to right (90 %).

However, the ratio m, reflecting vector control measures, has a major influence on the cumulative number of asymptomatic infections. For a scenario with no intervention, tripling the ratio to m = 3 results in a 911 % increase in the median cumulative number of asymptomatic infections, which is 2,600,865 (95 % CI: 5 - 2,688,653), equivalent to 69 % of the population. For a scenario with m = 6 and no interventions, the median cumulative number of asymptomatic infections after day 365 increases by 1050 % compared to m = 1. Increasing the delay before interventions generally causes the cumulative number of asymptomatic infections to increase. Lowering coverage impacts asymptomatic cases more than modest delays in starting interventions. In fact, using optimal vaccination parameters (minimal start-up delays and high coverage) can offset a slight rise in the m ratio. For example, when we compare a scenario with 10 % coverage and m = 1 against one with 90 % coverage and m = 3, both with a 7-day delay, we find in Fig. 2 that the total numbers of asymptomatic, severe, and fatal cases are similar. However, Fig. 3 reveals a timing difference: in the m = 3 scenario, case counts peak at the beginning of month 8, whereas with m = 1, they continue rising until the end of the simulation. This suggests that the cumulative case totals for m = 1 would grow even more if we extended the model beyond its current endpoint. With m = 3 and 90 % coverage, decreasing the 30-day delay to 14 days and 7 days produces a decrease in cases by 44 % and 56 %, respectively. This is smaller than the decrease in cases yielded by increasing vaccination coverage. The magnitude of the decrease resulting from a 30-day delay to a 7-day delay decreases as vaccination coverage decreases. To facilitate comparison across different transmission settings, the following paragraph presents corresponding baseline data (i.e., no vaccination) for m = 3 and m = 6, highlighting the influence of the mosquito-to-human ratio on disease burden.

#### 3.1.2 Severe infections

For severe infections, the median cumulative case count after day 365 for the baseline scenario (m = 1 and no intervention) is 44,459 (95 % CI: 1–165,008). This corresponds to 1.2 % of the population. When 10 % of the affected population is vaccinated and there is a 7-day delay, the median cumulative case count decrease 24 % in cases, while 14-day and 30-day delays decrease the cumulative case count by 18 % and 17 % compared to the baseline. As with the asymptomatic cases, these median case counts are similar, and the differences are due to stochastic variation. The upper limit follows the same increasing trend: 7-day, 14-day, 30-day, and no intervention. When m = 1, the effect of vaccination delay is obscured by the stochastic effects of the simulation. Increasing the vaccination coverage to 50 % and 90 % produces a significant decrease in the median case counts. For the scenario with a 7-day delay, the median case counts decrease by 70 % and 97 %.

When m is tripled to m = 3, the median case count increases by 1030 % compared to m = 1. For m = 6, the median case count increases by 1180 %. Increasing the delay causes the median cases to grow, but only by a small margin. For the scenario with m = 3, 30-day delay and 90 % coverage for the affected population, the median case count is 121,782 (95 % CI: 42,711 - 135,524). Decreasing the delay to 14 days and 7 days causes the median case count to fall by 44 % and 56 % respectively. The proportion of decrease is smaller for 10 % and 50 % coverage.

#### 3.1.3 Deaths

For deaths, the median number of cases for the baseline scenario was 8,162 (95 % CI: 0–35,951); this corresponds to 0.2 % of the total population. When 10 % of the affected population is vaccinated, and there is a 7-day delay, the median number of cumulative cases decreases by 22 %. With a 14-day delay and 30-day delay, the median cases fall by 17 % and 17 % respectively. Increasing the vaccination coverage to 50 % and 90 %, produces substantial decrease in the median cases. For scenario with a 7-day delay, the median number of cases for coverage 50 % and 90 % decreases by 68 % and 97 % respectively compared to baseline.

Tripling the ratio m to m = 3, the median number increases by 1370 % compared to baseline. For m = 6, the median number increase by 1610 %. Increasing the delay increases the median number by a small margin. For the scenario with m = 3, 30-day delay and 90 % coverage, the median number is 30,165 (95 % CI: 8933 - 33,372). Decreasing the delay to 14-day and 7-day causes the median death cases to fall by 44 % and 55 %. 10 % and 50 % coverage yield a smaller proportion of decrease.

#### 3.1.4 Overall comparisons of intervention variables

In general, decreasing the *m* ratio yields the most significant decrease in cumulative cases compared to increasing the vaccination coverage and decreasing the delay for asymptomatic infections, severe infections, and deaths. This is followed by increasing vaccination coverage and then decreasing vaccination delay. The effects of delay are negligible for *m* = 1. The impacts of vaccination coverage and delay on the number of cases are more apparent at a higher *m*. The delay also had a more significant effect with higher vaccination coverage.

Higher vaccination coverage significantly reduces transmission across all mixing scenarios. For example, with m = 3 and a 7-day delay, 90 % vaccination coverage reduces the median cumulative asymptomatic cases by 88 % compared to the no-vaccination baseline. Similarly, 50 % and 10 % coverage achieve reductions of 56 % and 12 %, respectively. These trends persist for m = 6, though with attenuated effects due to increased contact rates.

### 3.2 Active cases

Fig. 3 displays the time series of active cases for all stages for a fixed vaccine delay (delay = 7 days). The time series graphs for different delays, but the same vaccination coverage and m ratio configurations, have a similar shape to Fig. 3. For the susceptible stage, the number of susceptible people for m = 3 and m = 6 stabilizes before day 365 for all vaccination coverage levels considered. For 10 % and 50 % coverage in particular, the median number of susceptible people at day 365 for *m* = 3 was about 650,000. For *m* = 6, it was about 250,000. Roughly the same number of people are vaccinated or infected. For 90 % coverage, the median numbers of susceptible people for *m* = 3 and *m* = 6 are 341,416 and 161,846 respectively. For *m* = 1, the median numbers of people for 10 %, 50 %, and 90 % coverage are 3,045,382, 2,382,566 and 2,954,256, respectively.

Among all those exposed, the peak numbers of active cases for *m* = 6 are 258,098, 139,970 and 65,495 for 10 %, 50 % and 90 % coverage, respectively, reflecting a halving for each successive increase in coverage. Outbreaks peaked at Day 168, 168 and 161, respectively, around five and a half months since Day 1. The Full Width at Half Maximum (FWHM) values are 67, 73 and 55 days respectively.

For *m* = 3, the peak numbers of active cases are 160,082, 69,734 and 19,649 for 10 %, 50 % and 90 % coverage respectively. The percentage reductions are 56 % and 72 % for each successive increase in coverage. The days it peaks are Day 224, 238 and 218, respectively, about seven and a half months after Day 1. The FWHM values are 103, 126 and 100 days, respectively.

For *m* = 1, the number of active cases increased gradually for all coverage scenarios, highlighting that the vaccination strategies did not completely stop the spread of the disease but instead slowed transmission. The numbers of active cases on Day 365 are 46,958, 13,602 and 1,543 for 10 %, 50 % and 90 % coverage respectively.

### 3.3 Recovered cases

For the recovered stage, the total numbers of cases (this includes people who gain immunity from the vaccine) for m = 6 are similar for all vaccination coverages (3,391,185, 3,454,399 and 3,579,356 for 10 %, 50 % and 90 % coverage, respectively). This suggests that nearly everyone contracts yellow fever or receives vaccination. For *m* = 3, the total recovered cases are 2,952,328, 3,063,061 and 3,410,450. Lastly, for *m* = 1, the total recovered cases are 626,891, 1,360,276 and 814,413.

### 3.4 Vaccinations

**Fig. 4:**
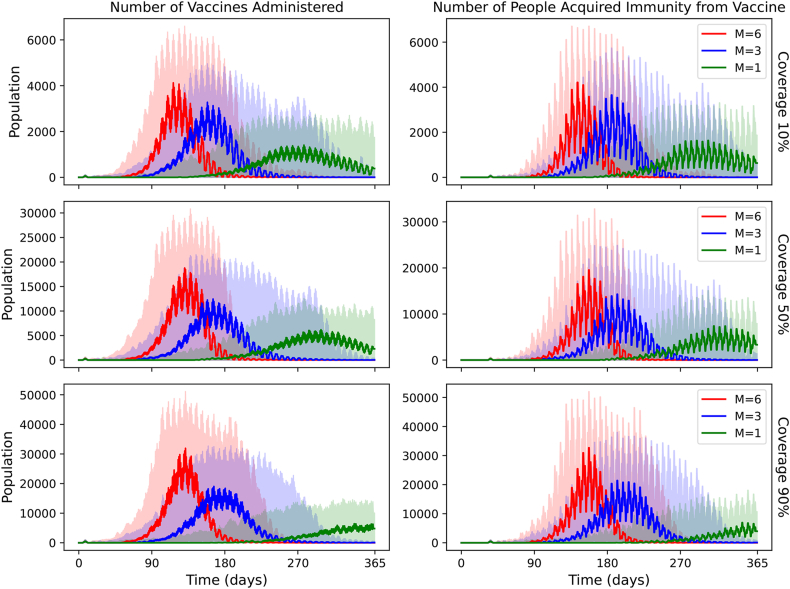
Daily vaccination doses and people who have gained immunity by vaccination, with a moving average of 3 days for different *m* scenarios and a fixed vaccine delay (delay = 7 days).

Fig. 4 shows the daily doses of vaccine administered in each scenario and the daily immunity gained. For *m* = 6, the vaccination curves peak at 4,124, 18,761 and 31,913 doses for 10 %, 50 % and 90 % coverage respectively. The days corresponding to the peak are Days 117, 131 and 132. The FWHM values are 44, 40 and 39 days. For *m* = 3, the vaccination curves peak at 3276, 12,437, and 19,036 doses, on Days 159, 166 and 167, respectively. The FWHM values are 57, 52 and 53 days. For *m* = 1, the vaccination curves peak at 1,405, 6182 and 6445 doses, on Days 278, 299 and 340, respectively. The FWHM values are 99 and 95 days.

**Fig. 5:**
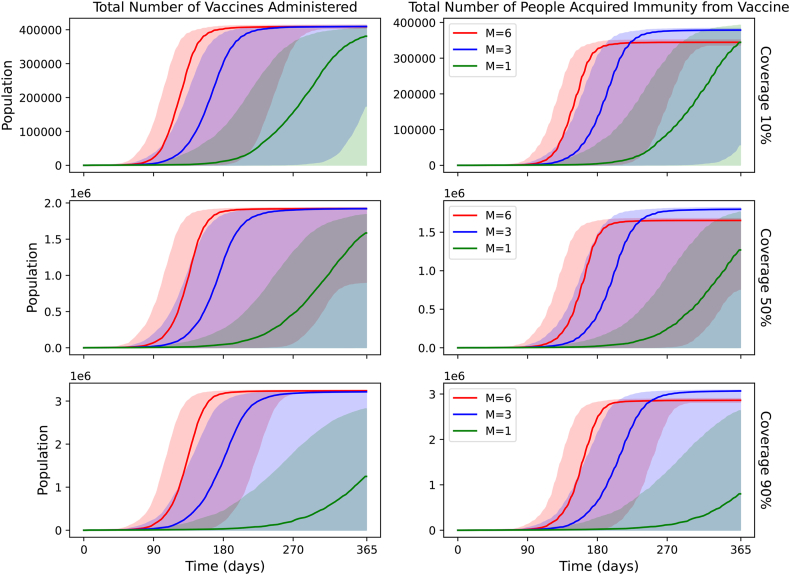
Cumulative vaccination doses and people who have gained immunity by vaccination, for different *m* scenarios and a fixed vaccine delay (delay = 7 days).

Fig. 5 shows the cumulative vaccine doses administered in each scenario and the cumulative immunity gained. For *m* = 3 or 6, the percentage of the population that has received the vaccine at the end of the simulation is nearly equal to the coverage. This suggests that for large *m*, extensive areas for ring vaccination or blanket vaccination would be required. For *m* = 6, we estimate that about 15 % of the population which is vaccinated would have already contracted immunity through a Yellow Fever infection.

### 3.5 Spatial distribution

**Fig. 6:**
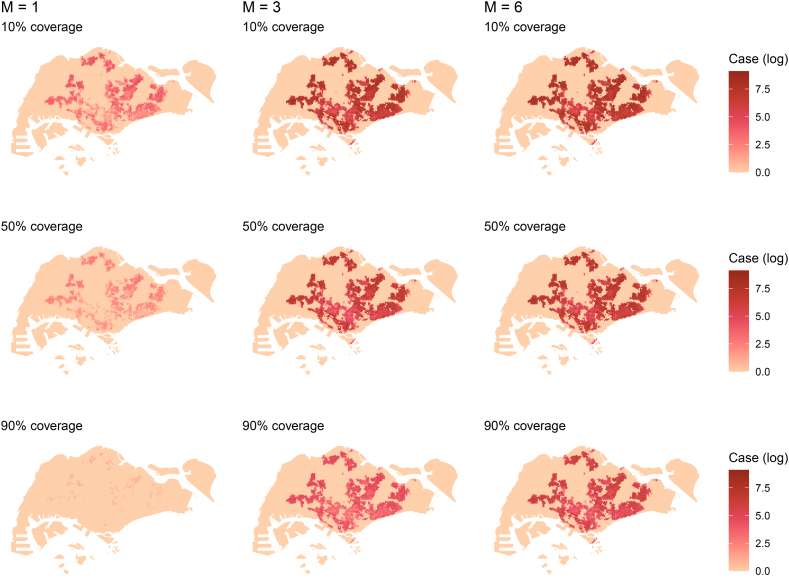
Spatial distribution of total number of exposed cases after Day 365 with log(1 + *x*) scale for different *m* ratios and vaccination coverage levels.

**Fig. 7:**
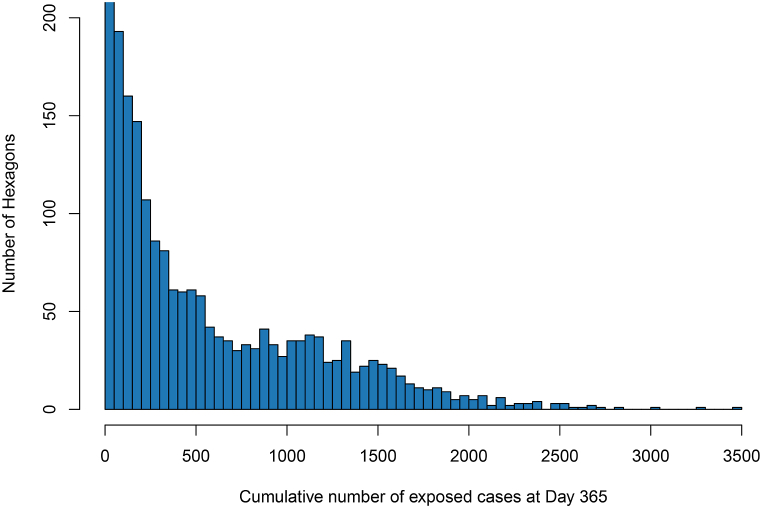
Histogram of the number of cumulative exposed cases after Day 365 with a bin width of 50 for *m* = 3, 7 days delay and 50 % coverage. The upper limit was set to 200 for the vertical axis.

For each hexagonal unit across the map, we computed the mean number of exposed cases across 100 independent simulation runs. Fig. 6 shows the spatial distribution of the total number of exposed cases after Day 365, averaged over all simulations with the same configuration. The spatial distribution of cases resembles that of the residential population. Fig. 7 shows the histogram of the cumulative number of cases in each hexagon for *m* = 3, a 7-day delay and 50 % coverage at Day 365. We fix the upper limit of the y-axis at 200 to better resolve the numbers of hexagons displayed. This results in the first bin (0-50) being cut off; the actual number of hexagons in that bin is 6,827, consisting mostly of hexagons with 0 cases. The number of hexagons falls exponentially as the case count increases, with relatively few hexagons having a case count of more than 2,000. It shows that most cases are concentrated in high-density regions.

### 3.6 Expected reproduction number

**Fig. 8:**
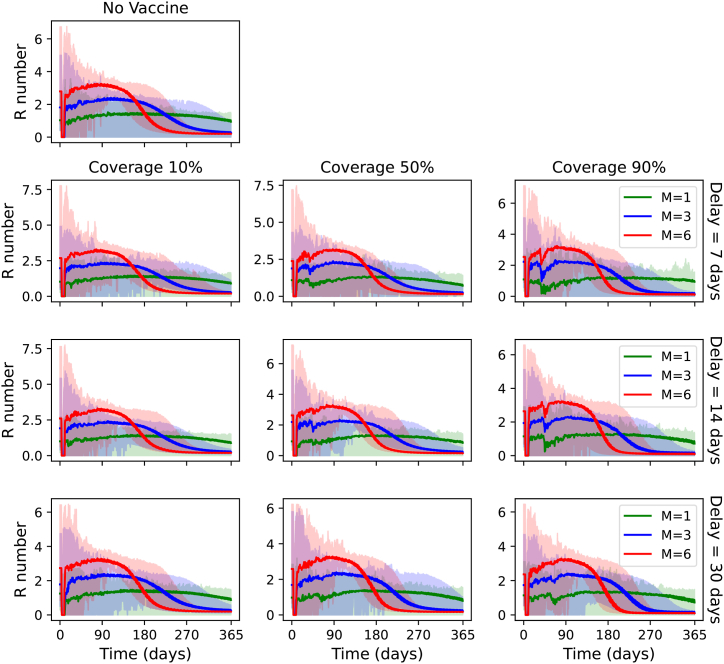
Combined spatial *R*_eff_ weighted by number of active cases.

Fig. 8 shows the evolution of expected *R*_eff_. The expected *R*_eff_ is calculated as a weighted average, where the weighting equals the sum of number of infected cases and number of asymptomatic cases multiplied by the expected relative infectivity E[*ψ*] = 0.3 (that is, *I*_*h*_ + E[*ψ*]*A*_*h*_). When comparing the scenarios with a 7-day delay, *m* = 6 and different coverage levels, we see that the inflexion point of *R*_eff_ coincides roughly with the peak of the exposed cases. This is because the gradient of *S*_*h*_ is equal to the gain in *E*_*h*_, and *R*_eff_ is proportional to *S*_*h*_. Similar observations can be made for *m* = 3. The inflexion point is also close to *R* = 1; this is because it becomes harder for *R* to further decrease (by means of vaccination) without more *S*_*h*_ to get infected (as vaccinations are only applied on transmission to new hexagons). For *m* = 1, the expected *R*_eff_ value hovers around *R* = 1, because the ring vaccination strategy only slows the transmission of YF, but transmission to new hexagons can still occur sporadically.

A dip in *R* occurs within the first 90 days for all scenarios with vaccination (disregarding the initial dip at the start of the simulation, which is an artefact of the simulation). The dip is more prominent with larger vaccination coverage and shorter delays. This is because of the first batch of vaccinations administered taking effect. After the dip, the *R* value remains lower than in the corresponding scenario with no vaccination.

### 3.7 Ring vaccination area

To investigate whether increasing the ring vaccination area (i.e. to include neighbouring hexagons for ring vaccination) aids in suppressing the spread of YF, we inspect the proportion of simulations containing at least 1 satellite outbreak after Day 3, 7, 10 and 14. Satellite outbreaks are outbreaks occurring at least 3 km away from the epicentre of the outbreak. Lower vaccination coverage (e.g., 10 %) results in fewer satellite outbreaks, as outbreaks grow into large, spatially continuous epidemics rather than isolated satellite events. Table 2 shows the proportion of simulations that have at least 1 satellite outbreak.

**Table 2:** Proportion of simulations with satellite outbreaks (SO) for Days 3, 7, 10 and 14. For instance, SO-3 denotes the proportion of simulations with satellite outbreaks on Day 3.

Scenario	SO-3 (%)	SO-7 (%)	SO-10 (%)	SO-14 (%)
*m* = 1, No vaccination	34	42	56	59
*m* = 1, *vc* = 10 %, *d* = 7	39	46	60	65
*m* = 1, *vc* = 10 %, *d* = 14	33	50	68	75
*m* = 1, *vc* = 10 %, *d* = 30	38	46	59	66
*m* = 1, *vc* = 50 %, *d* = 7	43	54	64	69
*m* = 1, *vc* = 50 %, *d* = 14	40	47	56	60
*m* = 1, *vc* = 50 %, *d* = 30	39	49	67	72
*m* = 1, *vc* = 90 %, *d* = 7	44	51	68	76
*m* = 1, *vc* = 90 %, *d* = 14	45	50	63	71
*m* = 1, *vc* = 90 %, *d* = 30	45	56	69	72
*m* = 3, No vaccination	44	54	75	81
*m* = 3, *vc* = 10 %, *d* = 7	33	46	67	72
*m* = 3, *vc* = 10 %, *d* = 14	45	56	73	82
*m* = 3, *vc* = 10 %, *d* = 30	36	38	56	65
*m* = 3, *vc* = 50 %, *d* = 7	37	45	60	69
*m* = 3, *vc* = 50 %, *d* = 14	36	49	76	79
*m* = 3, *vc* = 50 %, *d* = 30	37	43	59	68
*m* = 3, *vc* = 90 %, *d* = 7	31	49	63	70
*m* = 3, *vc* = 90 %, *d* = 14	35	40	64	72
*m* = 3, *vc* = 90 %, *d* = 30	34	43	61	62
*m* = 6, No vaccination	42	51	71	77
*m* = 6, *vc* = 10 %, *d* = 7	37	49	70	74
*m* = 6, *vc* = 10 %, *d* = 14	33	46	67	80
*m* = 6, *vc* = 10 %, *d* = 30	40	57	74	83
*m* = 6, *vc* = 50 %, *d* = 7	45	56	76	83
*m* = 6, *vc* = 50 %, *d* = 14	24	44	76	81
*m* = 6, *vc* = 50 %, *d* = 30	45	56	76	80
*m* = 6, *vc* = 90 %, *d* = 7	29	40	64	77
*m* = 6, *vc* = 90 %, *d* = 14	29	42	69	74
*m* = 6, *vc* = 90 %, *d* = 30	42	57	74	79

After 7 days, more than 50 % of simulations contain satellite outbreaks. Thus, for ring vaccination to be effective, short response time is crucial. Moreover, as it takes 21 days to reach full immunity after vaccination, it is important that hosts remain in their own hexagons to prevent the spread of YF. Furthermore, it is shown that increasing ring vaccination area is unlikely to significantly stop disease spread as most Singaporeans live and work at distinct locations. The average commuting distance between home and workplace often exceeds the radius used in ring vaccination strategies (Singapore Department of Statistics, 2021). This spatial separation allows individuals to transmit the virus in areas not covered by the vaccination ring. The details are in Appendix C.

## 4 Discussion

Given the presence of dengue, Zika and other mosquito diseases in Asia, it is unlikely that YF has never been introduced into Singapore. Several hypotheses have been proposed to explain the low incidence of YF in Asia. First, the high population seroprevalence of dengue and other flaviviruses may provide cross-protective immunity; studies have shown that dengue-immune individuals infected with YF are less likely to transmit YF to mosquitoes. Second, the dengue virus may outcompete YF within *Aedes aegypti*, thereby preventing co-infection (Wasserman et al., 2016). The ratio of female *Aedes aegypti* to humans for Singapore is estimated at 0.82 (Cracknell Daniels et al., 2021). Inputting expected values for parameters in Equation (17), the basic reproduction number, *R*_0_, is 1.19. Adding the effect of dengue could push the reproduction number below the critical value of 1. Third, YF has lower peak viral loads than dengue, limiting its transmission potential in dengue-endemic environments (Wasserman et al., 2016). Fourth, preventative measures may have played a role (Kuno, 2020). Many Asian countries require proof of YF vaccination for international travellers from countries with a high risk of YF. A previous study has estimated the probability of autochthonous transmission in Singapore at 0.05 (Cracknell Daniels et al., 2021).

Four main conditions for YF outbreaks have been suggested: sufficient migratory flow of viraemic individuals from YF endemic areas, a high density of competent vectors in receptive ecological conditions, a susceptible human population, and inadequate surveillance with delayed recognition leading to ineffective vaccination programmes (Wasserman et al., 2016). With warmer climates enhancing mosquito breeding and increased travel frequency between countries with endemic YF and Asia, concerns of potential YF outbreaks in Southeast Asian countries such as Singapore are increasingly pertinent (Lataillade et al., 2020). Given dengue is not hyperendemic in Singapore, Singapore is vulnerable to future YF outbreaks as dengue might not sufficiently suppress YF; this is true in many Asian countries. Despite stringent border controls in Singapore requiring proof of YF vaccination for travellers from YF endemic countries, asymptomatic but infectious cases may seed local YF outbreaks, necessitating pandemic preparedness (Cracknell Daniels et al., 2021).

Our results provide response times and levels of vaccine coverage needed to avert potentially severe YF infections and resulting deaths within local outbreak clusters. Our results demonstrate that a greater number of severe infections and deaths can be mitigated by decreasing the ratio *m* compared to ring vaccination strategies. The marginal gains in averting the number of infections and deaths are most significant when *m* is decreased, followed by increased vaccination coverage and reduced intervention delay as *R*_0_ is proportional to √*m*. This highlights the central role of vector control.

If vector control fails, however, vaccination can still suppress YF outbreaks. The number of averted cases via vaccination grows with the vector to human ratio *m*. Case count tends to decrease exponentially with vaccination coverage as seen in Fig. 4, so coverage as low as 50 % could still be considered. Decreasing the delay in outbreak identification had a minimal effect when compared to vaccination coverage and the *m* ratio.

Cities that are close to fulfilling all four conditions for a YF outbreak, such as in Southern China and Hong Kong, should stockpile YF vaccines. With concerns of mosquito population invasion and re-invasion, growing mosquito populations, and increasing global travel of infected individuals and mosquitoes, an outbreak of YF could occur in Asia in the future, making vaccination modelling critical. Cities in Asia have restrictions on travellers who have been in countries with endemic YF. Still, if YF begins to spread, border control may become increasingly challenging as 85 % of cases are asymptomatic and may be contracted in areas where YF has not yet been declared endemic.

We provided an estimate on the number of vaccine stockpiles required for Singapore. The parameters used here can be adapted to other similar cities. Using a conservative modelling framework for a potential yellow fever outbreak in Singapore, we estimate vaccine stockpile requirements under several key assumptions. First, without detailed local entomological data, the female-mosquito-to-human ratio (m) is set to 1, exceeding previous estimates of 0.82 in Singapore (derived from neighbouring country Malaysia) (Cracknell Daniels et al., 2021) and 0.17 pupae per person in Geylang (Seidahmed et al., 2018), to ensure a cautious planning scenario. A seven-day ring-vaccination delay is assumed, reflecting Singapore's efficient logistics and connectivity. Although 92 % of the population has completed primary vaccination against COVID-19 (Koh et al., 2024), only 50 % coverage is assumed here, ensuring that infants and those over 60, for whom the yellow fever vaccine is contraindicated, are excluded.

Simulations over one year indicate a median administration of approximately 1.6 million doses (about 50 % of the 3.3 million simulated population) by Day 365 under 50 % coverage. Interestingly, increasing initial coverage to 90 % produces a similar cumulative vaccine demand by year's end; bolstering early immunity primarily constrains outbreak dynamics later, rather than reducing total vaccine need.

Temporal analysis of vaccine uptake (Fig. 4) shows negligible demand in the first half of the year, with administration peaking around Month 10. This suggests that an immediate stockpile of the full one-year requirement is unnecessary. Instead, maintaining 25 % of the total population's worth of doses on hand should suffice to manage the outbreak through the first ten months, allowing real-time monitoring and additional ordering thereafter.

Ultimately, although fixed here at m = 1, the mosquito-to-human ratio will remain the principal determinant of outbreak size, and by extension, total vaccine demand, since the basic reproduction number R scales with $\sqrt{m}$ (refer to Appendix). More studies on m are encouraged as this significantly helps to accurately determine the stockpile required.

Our study has several limitations. Firstly, YF has not circulated locally in Singapore, and our spatial interaction model cannot be calibrated on observed case counts, unlike several other modelling studies looking into YF vaccination. However, epidemiological parameters were derived from prior studies, and uncertainties were incorporated by stochastic simulations. In the case of an outbreak, the experimental simulation can be enhanced with real-time surveillance data. We can incorporate new observations by re-weighting or resampling our ensemble of stochastic simulations according to how well each member predicted the incoming data. In practice, this is done with particle filtering (sequential Monte Carlo), which updates the distribution over hidden states in light of the new measurements. If we also have detailed data on regional disparities, such as mosquito abundance by region or willingness to vaccinate by district, we can apply a Bayesian update directly to the specific parameter of interest weighted by regional density (e.g. vaccine uptake probability for each hexagon) or unique parameters for each hexagon. After adjusting the hidden states and the model parameters, we use the revised ensemble as the starting point for the next forecast. Secondly, our model omits transmission between home and work or school, although this is likely insignificant as most Singaporeans use sheltered public or private transportation. Third, mosquitoes may disperse at distances beyond our hexagons' area (100,000 m^2^), particularly considering factors such as climate change (Explainer: How climate change is, 2022). Fourth, different locations may have a non-constant mosquito biting rate, so outbreak sizes of YF may significantly differ within a year. Singapore's climate is relatively consistent, so we do not consider this. Fifth, while the host population can be inferred from our data, the vector population is assumed to be in a constant proportion to the host population, and its exact distribution is difficult to obtain. Sixth, our model assumes a homogeneous population, although risk profiles may vary by average age of different estates. Seventh, our model only considers the scenario of local circulation from one importation event wherein a traveller infects local mosquitoes, whereas there could be multiple importation events. Eighth, there are uncertainties on the willingness to be vaccinated for YF, although, based on COVID-19 vaccination rates and the concerns surrounding a disease which could have severe complications, most of Singapore's residents may opt to be vaccinated (Griva et al., 2021; Shah et al., 2022). Ninth, multiple indirect effects from the interventions and modifications to human behaviour are likely, which are difficult to quantify and measure. Finally, numerous factors remain challenging to assess, such as increased infection/biting rates at mass gatherings, delay in individuals not seeking immediate treatment and the effects of community-wide behavioural shifts in response to knowledge of the outbreak.

## 5 Conclusion

Our modelling approach explores the impact of ring vaccination post-YF importation. We estimate large outbreak sizes for the vector to human population ratios explored. Vector control, therefore, remains critical alongside vaccination. The impact of vaccination is a substantial reduction in outbreak size proportional to the coverage level. Time-to-case detection was less critical but also moderated the outbreak size significantly. With the potential expansion of mosquito distributions driven by environmental factors (Colón-González et al., 2021), stockpiling of YF vaccines as part of outbreak preparedness should be considered.

## CRediT authorship contribution statement

**Guo Jing Yang:** Writing – review & editing, Writing – original draft, Validation, Methodology, Investigation, Formal analysis. **Haolong Song:** Methodology, Investigation. **Jue Tao Lim:** Methodology. **A. Janhavi:** Writing – review & editing. **Gregory Gan:** Writing – review & editing. **Guan Tong:** Writing – review & editing. **Pei Ma:** Methodology. **Nigel Lim Wei Han:** Writing – review & editing. **Muhammad Hafiz Bin Mohd Aziz:** Writing – review & editing. **Borame L. Dickens:** Writing – review & editing, Project administration, Methodology, Conceptualization.

## Ethical approval

Not applicable.

## Availability of data and materials

Data is available upon request.

## Funding

This work was supported by the Ministry of Health, Singapore, Population Health Metrics and Analysis (DEMOS) Grant E-608-0-0017-03 and PREPARE.

## Declaration of competing interest

The authors declare that they have no known competing financial interests or personal relationships that could have appeared to influence the work reported in this paper.

## Figures and Tables

**Table 3:** Mean and standard deviation of the distance between origin hexagon and furthest hexagon.

Scenario	Mean distance (m)	SD (m)
*m* = 1, No vaccination	3141	4600
*m* = 1, *vc* = 10 %, *d* = 7	4123	5692
*m* = 1, *vc* = 10 %, *d* = 14	3316	4839
*m* = 1, *vc* = 10 %, *d* = 30	3398	4713
*m* = 1, *vc* = 50 %, *d* = 7	3953	4820
*m* = 1, *vc* = 50 %, *d* = 14	4340	5571
*m* = 1, *vc* = 50 %, *d* = 30	3898	5266
*m* = 1, *vc* = 90 %, *d* = 7	4291	5600
*m* = 1, *vc* = 90 %, *d* = 14	4292	5487
*m* = 1, *vc* = 90 %, *d* = 30	4517	5706
*m* = 3, No vaccination	4591	5952
*m* = 3, *vc* = 10 %, *d* = 7	2802	4452
*m* = 3, *vc* = 10 %, *d* = 14	4770	6016
*m* = 3, *vc* = 10 %, *d* = 30	3760	5635
*m* = 3, *vc* = 50 %, *d* = 7	3432	4498
*m* = 3, *vc* = 50 %, *d* = 14	4016	5459
*m* = 3, *vc* = 50 %, *d* = 30	3743	5066
*m* = 3, *vc* = 90 %, *d* = 7	3984	6294
*m* = 3, *vc* = 90 %, *d* = 14	3162	4595
*m* = 3, *vc* = 90 %, *d* = 30	3876	5798
*m* = 6, No vaccination	3471	4493
*m* = 6, *vc* = 10 %, *d* = 7	3806	5630
*m* = 6, *vc* = 10 %, *d* = 14	3561	6086
*m* = 6, *vc* = 10 %, *d* = 30	4223	5300
*m* = 6, *vc* = 50 %, *d* = 7	4637	5498
*m* = 6, *vc* = 50 %, *d* = 14	2260	3619
*m* = 6, *vc* = 50 %, *d* = 30	4328	5589
*m* = 6, *vc* = 90 %, *d* = 7	3404	5554
*m* = 6, *vc* = 90 %, *d* = 14	3222	4916
*m* = 6, *vc* = 90 %, *d* = 30	3611	4708

**Table 4:** Cumulative case counts at Day 365 for exposed individuals and asymptomatic infections, with median cases and 95 % confidence interval in brackets.

Scenario	Exposed	Asymptomatic
*m* = 1, No vaccination	3313404 (3244313, 3352622)	496278 (484049, 502310)
*m* = 1, *vc* = 10 %, *d* = 7	386128 (5, 1229249)	44459 (1, 165008)
*m* = 1, *vc* = 10 %, *d* = 14	12011 (3, 58275)	1424 (1, 8003)
*m* = 1, *vc* = 10 %, *d* = 30	29734 (2, 70538)	3762 (1, 9470)
*m* = 1, *vc* = 50 %, *d* = 7	52412 (3, 147332)	6370 (1, 21416)
*m* = 1, *vc* = 50 %, *d* = 14	1346404 (14, 1447034)	197661 (2, 214390)
*m* = 1, *vc* = 50 %, *d* = 30	1424911 (964539, 1518758)	209072 (127880, 225432)
*m* = 1, *vc* = 90 %, *d* = 7	1744126 (217193, 1836863)	257716 (21124, 273554)
*m* = 1, *vc* = 90 %, *d* = 14	3167819 (2937290, 3203374)	474448 (425531, 480288)
*m* = 1, *vc* = 90 %, *d* = 30	3198852 (3086936, 3232852)	479382 (454576, 484035)
*m* = 3, No vaccination	2502424 (2439265, 2566784)	375099 (365350, 384197)
*m* = 3, *vc* = 10 %, *d* = 7	380344 (6, 937941)	45252 (1, 125522)
*m* = 3, *vc* = 10 %, *d* = 14	332972 (1, 1006274)	38463 (1, 135050)
*m* = 3, *vc* = 10 %, *d* = 30	380678 (2, 1012467)	45020 (1, 135759)
*m* = 3, *vc* = 50 %, *d* = 7	3077766 (5, 3173802)	456290 (1, 473431)
*m* = 3, *vc* = 50 %, *d* = 14	361374 (1, 395987)	53168 (1, 58697)
*m* = 3, *vc* = 50 %, *d* = 30	460354 (4, 499078)	68247 (1, 74533)
*m* = 3, *vc* = 90 %, *d* = 7	819262 (317855, 902851)	121782 (42711, 135524)
*m* = 3, *vc* = 90 %, *d* = 14	1876392 (502567, 1923695)	280759 (58702, 288201)
*m* = 3, *vc* = 90 %, *d* = 30	2018468 (1966566, 2068073)	302171 (292777, 309478)
*m* = 6, No vaccination	1690532 (1611247, 17940320)	253551 (241485, 269400)
*m* = 6, *vc* = 10 %, *d* = 7	111790 (1, 280359)	13410 (1, 37068)
*m* = 6, *vc* = 10 %, *d* = 14	73910 (0, 356711)	8832 (1, 47885)
*m* = 6, *vc* = 10 %, *d* = 30	143086 (4, 459939)	17112 (1, 63023)
*m* = 6, *vc* = 50 %, *d* = 7	2721532 (61176, 2809405)	401792 (4978, 417649)
*m* = 6, *vc* = 50 %, *d* = 14	2735480 (785274, 2825023)	404852 (97672, 420255)
*m* = 6, *vc* = 50 %, *d* = 30	2799418 (184375, 2902179)	414500 (16053, 432079)
*m* = 6, *vc* = 90 %, *d* = 7	3514352 (3468073, 3540101)	526539 (517191, 530808)
*m* = 6, *vc* = 90 %, *d* = 14	746840 (679474, 793038)	111624 (101327, 118665)
*m* = 6, *vc* = 90 %, *d* = 30	978052 (426456, 1046300)	146344 (62929, 156914)

**Table 5:** Cumulative case counts at Day 365 for severe infections and deaths, with median cases and 95 % confidence interval in brackets.

Scenario	Severe	Death
*m* = 1, No vaccination	123939 (119882, 125277)	2814990 (2750564, 2847610)
*m* = 1, *vc* = 10 %, *d* = 7	8162 (0, 35951)	285372 (4, 987085)
*m* = 1, *vc* = 10 %, *d* = 14	284 (0, 1826)	9012 (2, 47504)
*m* = 1, *vc* = 10 %, *d* = 30	750 (0, 2144)	22882 (2, 57229)
*m* = 1, *vc* = 50 %, *d* = 7	1250 (0, 5093)	39952 (2, 122820)
*m* = 1, *vc* = 50 %, *d* = 14	47992 (0, 52948)	1131418 (10, 1222227)
*m* = 1, *vc* = 50 %, *d* = 30	51110 (27072, 55590)	1195994 (770073, 1284511)
*m* = 1, *vc* = 90 %, *d* = 7	63298 (3265, 67655)	1473261 (147596, 1555454)
*m* = 1, *vc* = 90 %, *d* = 14	118388 (100707, 119834)	2690932 (2454072, 2722444)
*m* = 1, *vc* = 90 %, *d* = 30	119487 (110746, 121049)	2717178 (2602539, 2747282)
*m* = 3, No vaccination	93561 (90559, 96171)	2124828 (2070949, 2180678)
*m* = 3, *vc* = 10 %, *d* = 7	8740 (0, 27833)	285637 (5, 750975)
*m* = 3, *vc* = 10 %, *d* = 14	7124 (0, 29748)	246241 (1, 806855)
*m* = 3, *vc* = 10 %, *d* = 30	8788 (0, 29819)	286268 (2, 811271)
*m* = 3, *vc* = 50 %, *d* = 7	112180 (0, 117479)	2600864 (5, 2688653)
*m* = 3, *vc* = 50 %, *d* = 14	13052 (0, 14540)	303898 (1, 334738)
*m* = 3, *vc* = 50 %, *d* = 30	16800 (0, 18444)	388702 (4, 422372)
*m* = 3, *vc* = 90 %, *d* = 7	30164 (8933, 33372)	692802 (257731, 765385)
*m* = 3, *vc* = 90 %, *d* = 14	69958 (10100, 72008)	1592690 (377948, 1632885)
*m* = 3, *vc* = 90 %, *d* = 30	75279 (72101, 77246)	1713382 (1665780, 1756830)
*m* = 6, No vaccination	63390 (59972, 67507)	1437056 (1369190, 1524174)
*m* = 6, *vc* = 10 %, *d* = 7	2581 (0, 8105)	83746 (1, 223037)
*m* = 6, *vc* = 10 %, *d* = 14	1674 (0, 10430)	54960 (0, 284590)
*m* = 6, *vc* = 10 %, *d* = 30	3326 (0, 14191)	108163 (3, 372164)
*m* = 6, *vc* = 50 %, *d* = 7	98702 (671, 103548)	2296956 (37240, 2376374)
*m* = 6, *vc* = 50 %, *d* = 14	99324 (18898, 104231)	2309238 (607583, 2392532)
*m* = 6, *vc* = 50 %, *d* = 30	101786 (2085, 107405)	2361198 (118044, 2457123)
*m* = 6, *vc* = 90 %, *d* = 7	131484 (127796, 132774)	2985606 (2938935, 3008071)
*m* = 6, *vc* = 90 %, *d* = 14	27856 (25219, 29636)	633932 (576088, 673552)
*m* = 6, *vc* = 90 %, *d* = 30	36459 (15355, 39251)	830864 (358495, 888859)

**Fig. A.2:**
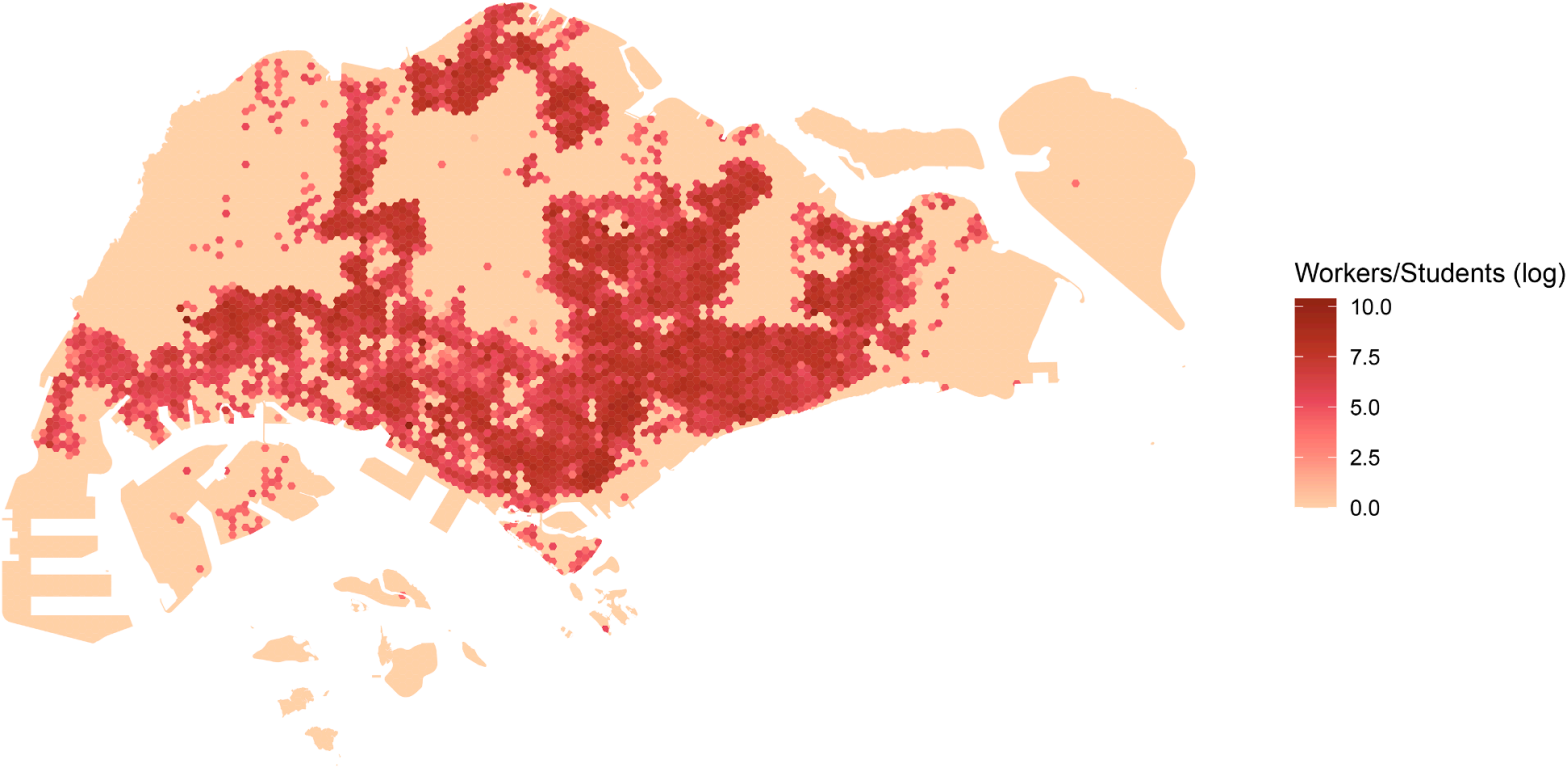
Workers' and students' population density in Singapore in 2023, with log(1+*x*) scale. The two outliers in the plot are the National University of Singapore and Nanyang Technological University.

## Appendix A Methods

### 1.1 Reproduction Number

The expression for the Basic Reproduction Number, *R*_0_, is

(17) $$R_{0}=\sqrt{\left(\psi +\delta -\psi \delta \right)\cdot \frac{a^{2}bcm}{\gamma_{h}}\cdot \frac{\sigma_{v}}{\mu_{v}\left(\sigma_{v}+\mu_{v}\right)}}$$

The description of parameters can be found in Table 1. The derivation of this expression is provided in Appendix B. The Effective Reproduction Number, *R*_eff_ is

(18) $$R_{\text{eff}}=R_{0}\cdot \frac{S_{h}}{S_{h}+E_{h}+A_{h}+I_{h}+T_{h}+R_{h}}$$

The rate *γ*_*h*_ is time-dependent as the host moves between hexagons. The second term in *R*_eff_ is also time-dependent due to stage progression (susceptible individuals become exposed).

### 1.2 Modelling

#### 1.2.1 Initialization

Each host starts in their home hexagon with unique host parameters (*σ*_*h*_^−1^, *γ*_*h*_^−1^, *θ*, *κ*_*h*_^−1^) uniformly generated from the range given in Table 1. The number of vectors in each hexagon was initialised by multiplying *m* with the number of hosts in the hexagon.

The ratio *m* is a fixed global parameter. Vectors (mosquitoes) within the same hexagon have the same vector parameters (*σ*_*v*_^−1^, *μ*_*v*_^−1^, *a*, *b*, *c* and *ψ* uniformly generated from the range in Table 1).

This is based on our assumption that each hexagon has unique environmental factors which provide random variation (Zahid et al., 2023).

#### 1.2.2 Configurations

We start each simulation with a scenario in which one host was infected, and the host had successfully transmitted YF to a vector in the hexagon the host lived in. The one infected host is randomly selected from the whole population. This assumption is based on another study which estimated the number of introductions of Yellow Fever in Singapore in 2016 to be 1. To incorporate the effect of movement in our simulation, the host alternates between the home and work/school hexagons. During weekdays, the host spends the first half of the day in their work/school hexagon and the remaining half in their home hexagon. On weekends, they stay in their home hexagon. If the host is infected during the simulation, the host returns to their home hexagon until they recover. Once the host returns to the home hexagon, any vector residing in the hexagon will have a chance of biting the infected host. If any vector becomes infectious after biting the infected host, the vector can spread the disease to anyone who is in the home hexagon (including people who have the same work hexagon as the infected host's home hexagon). Vectors remain in their hexagons throughout the simulation. Some studies outside Singapore suggested the dispersal distance to be greater than the size of the hexagon (Bogojević et al., 2011; David et al., 2009); we seek to capture such effects by the simulated movement of the host. Mosquitoes also tend to have a positive drift towards humans and breeding sites (usually residential areas), suggesting the dispersal distance is not necessarily much larger.

#### 1.2.3 Algorithms

We used different modelling approaches for the host and vector. For the host, we used agent-based modelling to account for the host's movement. For the vector, we used the classic SEI epidemic model.

The host and vector transition models interact in the transition between the susceptible and the exposed stages. For each individual in the host model, the transition from the susceptible to the exposed stage is determined by a Bernoulli trial, wherein the probability of success, *p*, is *abI*_*v*_/*N*_*h*_. At the exposed stage, each individual's transition pathway is determined by the probability *δ*. For the first pathway (Equation (1)), the probability of death is *θ*.

For the vector model, the ODEs are implemented with the predictor-corrector method. The explicit Euler method is used as a predictor, and the implicit second-order Adams-Moulton method (AM2) is used as a corrector. This ensures the overall errors are second-order at each step. In each time step, we track the location of all hosts and the infection stages for hosts and vectors.

For each configuration, we ran 100 independent simulations.

## Appendix B Basic Reproduction Number, *R*_0_

The basic reproduction number, *R*_0_, for the vector-host model, can be calculated from the rate of transmission vector F and rate of transition vector V (Brauer et al., 2016; Van Den Driessche & Watmough, 2002).

The rate of transmission vector F is given by

(19) $$F=\left(\begin{array}{c} \frac{ab}{N_{h}}I_{u}S_{h}\\ 0\\ 0\\ \frac{ac}{N_{h}}\beta_{h}A_{h}+I_{h}S_{v}\\ 0 \end{array}\right)$$

The rate of transition vector V is given by

(20) $$V=\left(\begin{array}{c} \sigma_{h}E_{h}\\ \left(\delta -1\right)\sigma_{h}E_{h}+\gamma_{h}A_{h}\\ -\delta \sigma_{h}E_{h}+\gamma_{h}I_{h}\\ -\left(\sigma_{v}+\mu_{v}\right)E_{v}\\ -\sigma_{v}E_{v}+\mu_{v}I_{v} \end{array}\right)$$

Under the disease-free equilibrium condition, we can derive the Jacobian *F* of F and Jacobian *V* of V:

(21) $$F=\left(\begin{array}{cccc} 0 & 0 & 0 & \text{ab}\\ 0 & 0 & 0 & 0\\ 0 & \text{ac}\gamma m & \text{acm} & 0\\ 0 & 0 & 0 & 0 \end{array}\right)$$

where *F*_i,j_ = ∂*F*_i_/∂*x*_j_ with *F*_i_ the i-th component of F and *x*_j_ is the j-th component of the vector (*E*_h_, *A*_h_, *I*_h_, *E*_v_, *I*_v_), and

(22) $$V=\left(\begin{array}{ccccc} \sigma_{h} & 0 & 0 & 0 & 0\\ \left(\delta -1\right)\sigma_{h} & \gamma_{h} & 0 & 0 & 0\\ -\delta \sigma_{h} & 0 & \gamma_{h} & 0 & 0\\ 0 & 0 & 0 & \sigma_{h}+\mu_{v} & 0\\ 0 & 0 & 0 & -\sigma_{v} & \mu_{v} \end{array}\right)$$

where *V*_i,j_ = ∂*V*_i_/∂*x*_j_, with *V*_i_ is the i-th component of V and *x*_j_ is the j-th component of the vector (*E*_h_, *A*_h_, *I*_h_, *E*_v_, *I*_v_). Then the next-generation matrix of our model is

(23) $$FV^{-1}=\left(\begin{array}{cccc} 0 & 0 & 0 & \frac{\text{ab}\sigma_{v}}{\mu_{v}\left(\sigma_{v}+\mu_{v}\right)}\\ 0 & 0 & 0 & \frac{\text{ab}}{\mu_{v}}\\ \frac{\text{acm}}{\gamma_{h}}\left(\psi +\delta -\psi \delta \right) & 0 & \frac{\text{acm}}{\gamma_{h}}\cdot \psi & 0\\ 0 & 0 & 0 & 0 \end{array}\right)$$

Since *R*_0_ is the spectral radius of *FV*^−1^, the basic reproduction number is

(24) $$R_{0}=\sqrt{\left(\psi +\delta -\psi \delta \right)\cdot \frac{a^{2}bcm}{\gamma_{h}}\cdot \frac{\sigma_{v}}{\mu_{v}\left(\sigma_{v}+\mu_{v}\right)}}$$

where *m* is the ratio of vector to human populations.

## Appendix C Distance from furthest outbreak to epicentre at Day 3

We inspect the distance from the centroid of the furthest hexagon to that of the hexagon of the YF epicentre after Day 3. The distance is considered zero if the outbreak does not spread to any other hexagon than that of the origin. Table 3 shows the mean and variance of this distance.

The standard deviation of all outbreaks' distances from the initial outbreak's hexagon was around 5 km. The distance distributions were positively skewed, with several simulations having no spread in the outbreak and a few simulations inducing an outbreak at about 20 km. These results were expected as our models only considered workplace and home transmission, not transmission in between these locations. As most Singaporeans live and work at distinct locations, our results suggest that increasing ring vaccination area coverage is unlikely to significantly stop disease spread.

## Appendix D Total number of cases at Day 365

Table 4 shows the median number of cases (with 95 % confidence interval) for exposed individuals and asymptomatic infections for all scenarios.

Table 5 shows the median number of cases (with 95 % confidence interval) for severe infections and deaths for all scenarios.
